# Racial Disparities in Survival of Breast Cancer Patients After Surgery

**DOI:** 10.3389/fpubh.2022.831906

**Published:** 2022-05-13

**Authors:** Shuhan Wang, Weifang Tang, Shengying Wang, Shikai Hong, Jianjun Liu

**Affiliations:** ^1^Breast Cancer Center, West District of The First Affiliated Hospital of the University of Science and Technology of China, Hefei, China; ^2^Division of Life Sciences and Medicine, University of Science and Technology of China, Hefei, China

**Keywords:** racial disparities, breast conserving surgery, breast cancer, incidence, survival

## Abstract

**Introduction:**

The racial disparities of opportunity to receive the appropriate intervention and lower insurance coverage may result in survival disparities in different races. This study aims to provide a perspective on racial disparities in the survival of breast cancer patients after surgery.

**Methods:**

Through data from the Surveillance, Epidemiology, and End Results (SEER) program, this study estimated the survival of breast cancer patients of different races from 1998 to 2017. Inverse probability weighting (IPW) was utilized to adjust the imbalanced clinicopathological features of patients of different races.

**Results:**

This study analyzed 214,965 breast cancer patients after surgery. Among them, 130,746 patients received BCS, and the remaining 84,219 breast cancer patients underwent mastectomy. Although Asian or Pacific Islander (API) patients after surgery showed higher survival benefit than that of white patients in the primary data, after adjusting for age at diagnosis, luminal subtype, grade, T stage, and N stage in different races, white individuals had the longest period of survival was higher than that of the minority groups in BCS group [breast cancer-specific survival (BCSS): HR_Whitevs.API_ = 0.402, HR_Whitevs.Black_ = 0.132; *P* < 0.001; overall survival (OS): HR_Whitevs.API_ = 0.689, HR_Whitevs.Black_ = 0.254; all *P* < 0.001] and mastectomy group (BCSS: HR_Whitevs.API_ = 0.325, HR_Whitevs.Black_ = 0.128; *P* < 0.001; OS: HR_Whitevs.API_ = 0.481, HR_Whitevs.Black_ = 0.206; all *P* < 0.001)

**Conclusions:**

We first identified that the survival benefit of the minority group after surgery was lower than that of white individuals, regardless of tumor chrematistics and surgery types.

## Introduction

In 2020, breast cancer was the highest incidence and the second leading cause of cancer death. It was estimated that in 2020, about 276,480 women or 30% of female cancer patients were diagnosed with breast cancer, and 42,170 cancer-related deaths were caused by breast cancer (1, 2). Racial disparities in tumor incidence and survival rates have been increasingly observed recently (3). For example, compared with black breast cancer patients, white breast cancer patients were more likely diagnosed at earlier stage (4). Nowadays, several studies have identified that not only racial differences in tumor pathological characteristics, but also the receipt of systemic therapy can lead to the survival disparities of breast cancer patients (4, 5). Breast cancer is a multifactorial disease, the survival disparities of breast cancer patients are also affected by other factors beyond the tumor stage, including system therapy after surgery and socioeconomic status (6).

There have been remarkable advances in the field of breast cancer surgery in the past 50 years (7). Currently, BCS has become a standard treatment for patients with early-stage breast cancer (8). Compared with mastectomy, patients receiving BCS rely on precise imaging, detailed pathology, specialist surgery during surgery, and systemic therapy after surgery to enhance survival (9). However, compared with white breast cancer patients, minority patients have less access to effective care and lower insurance coverage (10). These racial disparities may lead to survival disparities in survival in breast cancer patients.

Ongoing nationally funded research on racial disparities has led to new concerns about human health (11). In fact, understanding racial disparities is beneficial for doctors to select an effective treatment for patients from different races. In the current study, we will provide a clear perspective on racial disparities in the survival of breast cancer patients after surgery by analysis of a multirace dataset, which contributes to the formulation of targeted public health interventions and policies.

## Materials and Methods

### Survival Cohort

This study utilized the population-based SEER program to collect survival data for further analysis. Before analysis, we selected female patients with M0 (no distant organ metastasis) from 1998 to 2017 because information on the molecular subtypes of breast cancer patients were provided after 1998. All patients undergoing surgery (RX sum-Surg Prim Site code: 20–39 were regarded as BCS) were included and analyzed. As American Indian (AI) and Alaska Native patients have a low incidence of breast cancer, the sample of AI breast cancer patients undergoing surgery was too small for accurate survival analysis. Thus, this study excluded the survival of AI breast cancer patients to minimize heterogeneity.

### Statistical Analysis

Overall survival and BCSS were the main outcomes of this study. OS was defined as the algorithmic analysis of death, while BCSS referred to death caused by breast cancer. Other-cause mortality (OCM) was recorded as death caused by other diseases. OS and BCSS were calculated by the Kaplan–Meier method. Hazard ratios (HRs) were used for pairwise comparison.

Before pairwise comparison, this study was conducted stepwise IPW by a propensity model to adjust the baseline imbalance of patients from different races (12). IPW models were performed as follows: First, the age-adjusted IPW was only adjusted for the age imbalance at diagnosis. Second, age at diagnosis and lumen subtypes were adjusted, weighting the imbalance in age at diagnosis and luminal subtype. Third, IPW was fully adjusted, weighting the potential risk factors of the SEER program completely, including age at diagnosis, luminal subtype, grade, T stage, and N stage. Among these propensity IPW models, age at diagnosis was regarded as a continuous variable, and other factors were treated as categorical variables.

A *P*-value lower than 0.05 on both sides was considered statistically significant. Data were analyzed by version 3.4.3 of R software (R Foundation for Statistical Computing) and version 22 of Statistical Product and Service Solutions (SPSS).

## Results

### Patient Characteristics

**Figure 1:**
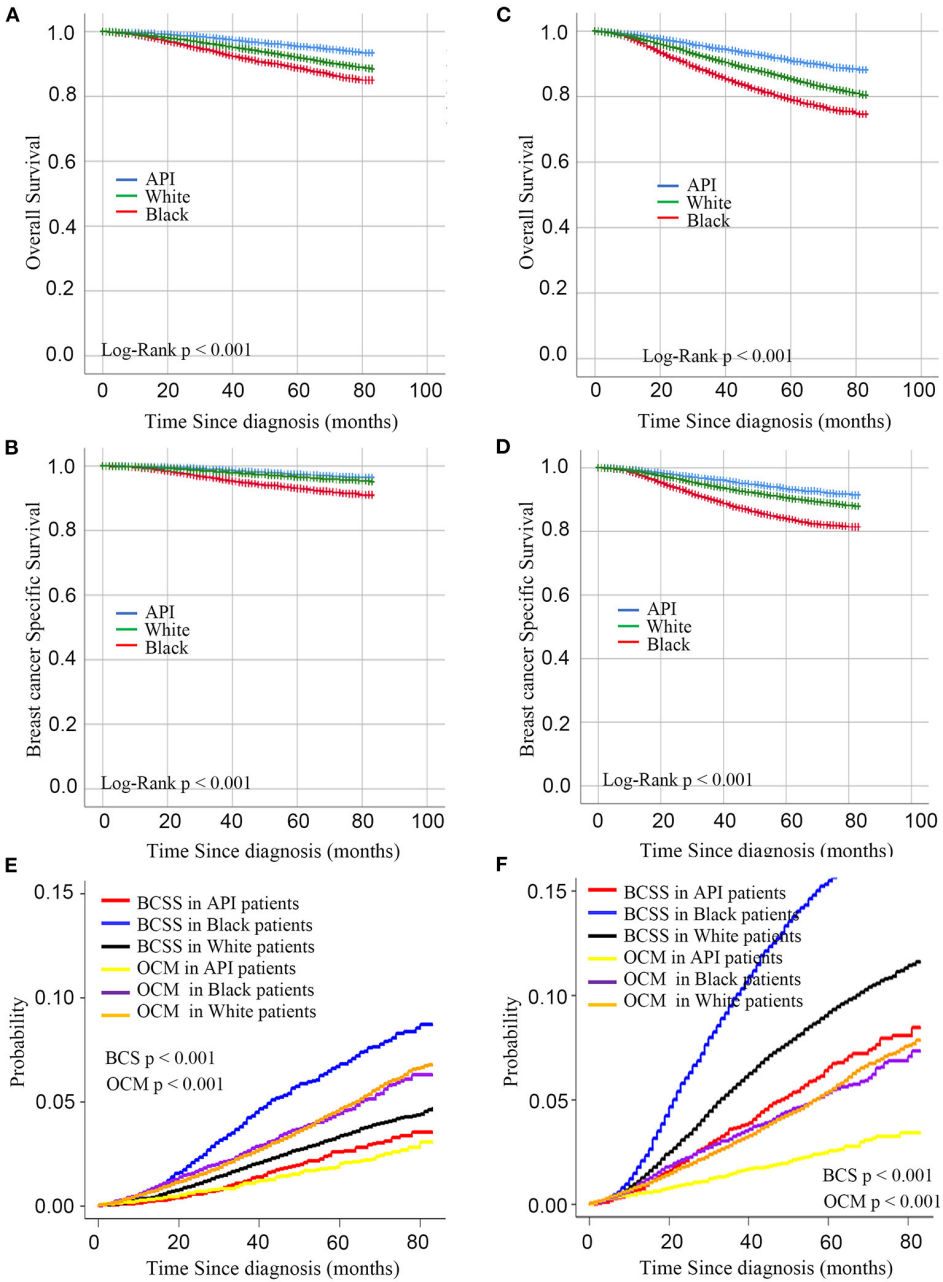
Kaplan– curves for overall survival and breast cancer-specific survival by race in the BCS group **(A,B)** and mastectomy group **(C,D)**. **(C)** Cumulative incidence function curves for racial disparities of breast cancer-specific death and other-cause mortality in BCS group **(E)** and mastectomy **(F)**.

As shown in Tables 1, 2, this study contained 214,965 breast cancer patients after surgery based on all criteria. Among them, a total of 130,746 breast cancer patients underwent BCS. 84,219 breast cancer patients underwent mastectomy. There were 171,799 white patients, 23,371 black patients, and 19,795 API patients. In the BCS group, black patients with breast cancer after BCS were likely to suffer from poorly differentiated or undifferentiated tumors, with a percentage of 43.63%. White patients tended to be infected with well-differentiated tumors, with a percentage of 29.61%. In addition, black patients with breast cancer had higher rates of TNBC or HER2-positive tumors, with a percentage of 25.28%. Compared with white and black patients, it was less likely for API patients to be diagnosed at T4 and N3 stages. As shown in the Figure 1, in the main dataset, the 5-year BCSS in white, black, and API patients was 96.9, 93.1, and 97.4%, respectively, and the 5-year OS in these three groups showed similar disparities at 95.5, 88.9, and 91.9%, respectively. In the mastectomy group, the 5-year BCSS in white, black, and API patients was 90.6, 83.9, and 84.1%, respectively, and the 5-year OS in these three groups showed similar disparities at 85.4%, 79.2, and 91.0%, respectively.

**Table 1:** Description of baseline characteristics of breast cancer patients underwent BCS.

	**White** ***n*** **= 106,200**		**Black** ***n*** **= 13,693**		**API** ***n*** **= 10,853**	
	**Mean ±SD/NO**.	**Percent (%)**	**Mean ±SD/NO**.	**Percent (%)**	**Mean ±SD/NO**.	**Percent (%)**
Age (year)	62.18 ± 12.30		59.02 ± 12.13		57.93 ± 12.42	
**Grade**						
Well-differentiated	31,444	29.61	2,468	18.02	2,815	25.94
Moderately differentiated	47,333	44.57	5,251	38.35	4,938	45.50
Poorly differentiated	27,206	25.62	5,947	43.43	3,072	28.31
Undifferentiated	217	0.20	27	0.20	28	0.26
**T stage**						
T1	78,707	74.11	8,720	63.68	7,637	70.37
T2	25,199	23.73	4,470	32.64	2,983	27.49
T3	1,858	1.75	409	2.99	191	1.76
T4	436	0.41	94	0.69	42	0.39
**N stage**						
N0	84,870	79.92	10,052	73.41	8,668	79.87
N1	17,963	16.91	2,938	21.46	1,868	17.21
N2	2,455	2.31	517	3.78	240	2.21
N3	912	0.86	186	1.36	77	0.71
**Luminal subtype**						
Luminal A	84,555	79.62	8,860	64.70	8,419	77.57
Luminal B	9,123	8.59	1,372	10.02	1,134	10.45
TNBC	9,496	8.94	2,841	20.75	843	7.77
HER2-positive	3,026	2.85	620	4.53	457	4.21
**Radiation therapy**						
Yes	79,425	74.79	10,141	74.06	8,088	74.52
NO/Unknown	26,775	25.21	3,552	25.94	2,765	25.48
**Chemotherapy**						
Yes	34,033	32.05	6,623	48.37	3,891	35.85
NO/Unknown	72,167	67.95	7,070	51.63	6,962	64.15
**Insurance**						
Insured	103,567	97.52	13,189	96.32	10,590	97.58
Uninsured/NA	2,633	2.48	504	3.68	263	2.42
Follow-up (months)	42.25 ± 21.31		41.31 + 20.98		41.14+ 21.57	

*APIs, asian or pacific islanders; TNBC, triple-negative breast cancer; NA, not available*.

**Table 2:** Description of baseline characteristics of breast cancer patients underwent mastectomy.

	**Whites** ***n*** **= 65,599**		**Blacks** ***n*** **= 9,678**		**APIs** ***n*** **= 8,942**	
	**Mean ±SD/NO**.	**Percent (%)**	**Mean ±SD/NO**.	**Percent (%)**	**Mean ±SD/NO**.	**Percent (%)**
Age (year)	57.87 ± 14.03		56.05 ± 12.13		55.98 ± 13.38	
**Grade**						
Well	11,437	17.40	1,123	11.60	1,316	14.70
Moderately	29,255	44.60	3,583	37.00	3,972	44.40
Poorly	24,660	37.60	4,941	51.10	3,613	40.40
Undifferentiated	247	0.40	31	0.30	41	0.50
**T stage**						
T1	29,793	45.40	3,709	38.30	4,001	44.70
T2	25,601	39.10	3,982	41.10	3,765	42.10
T3	7,242	11.00	1,349	13.90	839	9.40
T4	2,963	4.50	638	6.60	337	3.80
**N stage**						
N0	35,735	54.50	4,615	47.70	5,131	57.40
N1	20,173	30.80	3,292	34.00	2,617	29.30
N2	6,054	9.20	1,122	11.60	784	8.80
N3	3,637	5.50	649	6.70	410	4.60
**Luminal subtype**						
Luminal A	45,288	69.00	5,622	58.10	6,028	67.40
Luminal B	8,466	12.90	1,268	13.10	1,271	14.20
TNBC	8,043	12.30	2,105	21.80	892	10.00
HER-2 enriched	3,802	5.80	683	0.71	751	8.40
**Radiation therapy**						
Yes	17,805	27.10	3,305	34.10	2,234	25.00
NO/Unknown	47,794	72.90	6,373	65.90	6,708	75.00
**Chemotherapy**						
Yes	35,252	53.70	6,159	63.60	4,895	54.70
NO/Unknown	30,347	46.30	3,519	36.40	4,047	45.30
**Insurance**						
Insured	63,728	97.10	9,190	95.00	8,700	97.30
Uninsured/NA	1,871	2.90	488	5.00	242	2.70
Follow up (months)	42.76 ± 21.42		41.18 ± 21.17		41.09 ± 21.71	

*APIs, asian or pacific islanders*.

### Survival Analysis in BCS Group

**Figure 2:**
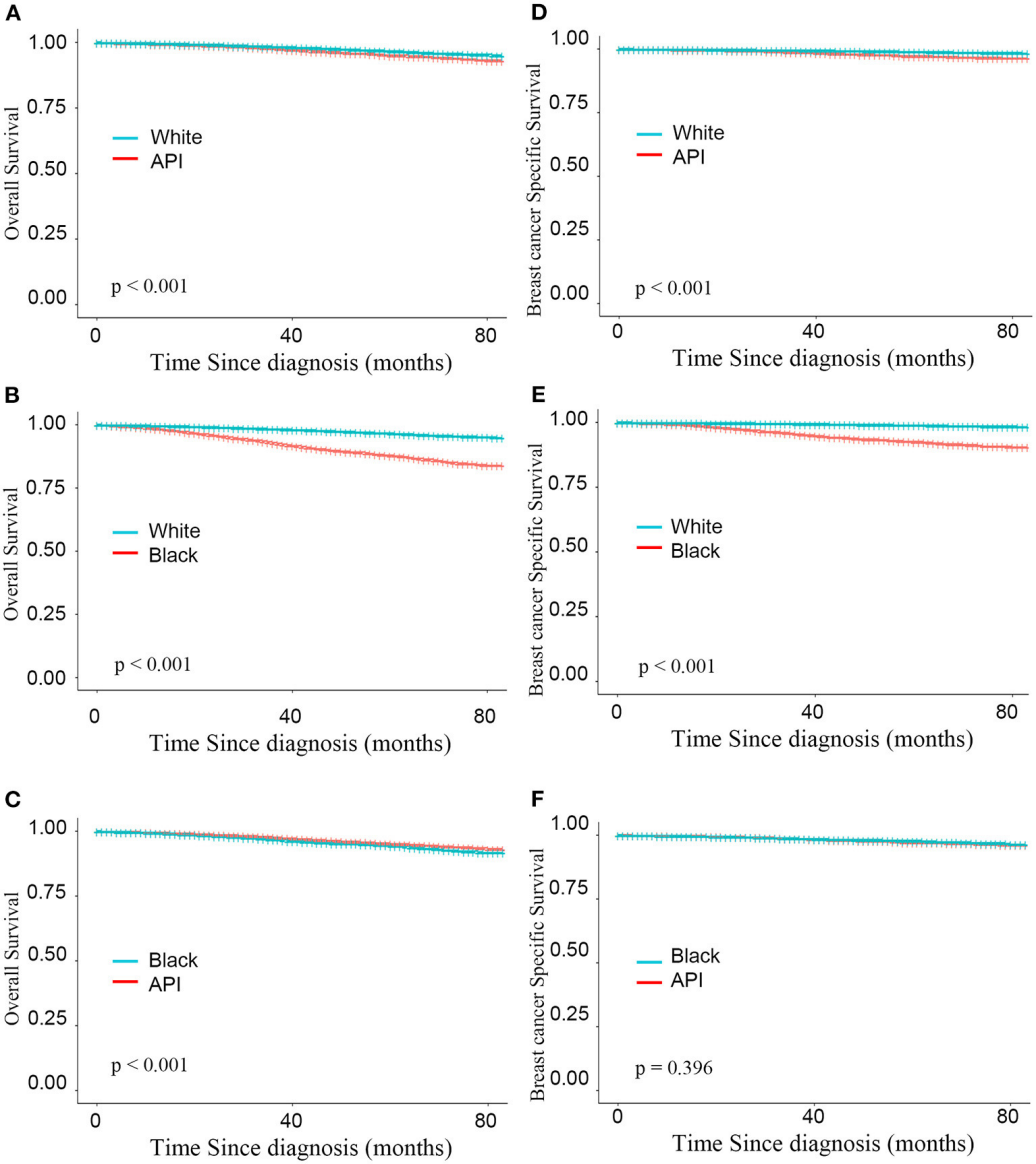
Kaplan–Meier curves for overall survival by race **(A–C)** and breast cancer-specific survival **(D–F)** based on the inverse probability weighting of all factors in BCS group.

A stepwise IPW method for sensitivity analysis in the primary dataset was performed in this study to understand the association between baseline characteristics and patient survival. First, based on the weighted imbalance of age during diagnosis, the subdistribution hazard ratio (SHR) of BCSS between white and API patients declined by 6.7% (from 1.440 to 1.373). In addition, white patients had a 74.8% reduction in the subdistribution OS hazard in the OS IPW model (from 1.892 to 1.144). In IPW models adjusted by age and luminal subtypes, survival differences between white and API patients did not disappear in either the BCSS or OS model (BCSS: HR = 0.999, CIs: 0.851–1.721, *P* = 0.987; OS: HR = 0.952, CIs: 0.843–1.077, *P* = 0.436). Compared with other races, white patients had the best BCSS and OS outcomes (as shown in Figure 2) after adjusting for an imbalanced baseline, but the survival advantages of API patients disappeared. There was no significant survival difference in black and API patients (BCSS: HR = 0.916, 0.749–1.121, *P* = 0.396) according to the subdistribution hazard model. Detailed SHR results are given in Table 3.

**Table 3:** Inverse probability weighted (IPW) estimates of BCSS and OS in patients underwent mastectomy.

	**BCSS**			**OS**		
	**HR**	**CIs**	***P***	**HR**	**CIs**	***P***
**Unadjusted**						
White vs. Black	0.473	0.434 to 0.516	<0.001	0.674	0.631 to 0.719	<0.001
White vs. API	1.440	1.230 to 1.686	<0.001	1.892	1.685 to 2.125	<0.001
Black vs. API	3.042	2.564 to 3.611	<0.001	2.807	2.470 to 3.190	<0.001
**Age-adjusted**						
White vs. Black	0.457	0.419 to 0.499	<0.001	0.428	0.398 to 0.46	<0.001
White vs. API	1.373	1.172 to 1.608	<0.001	1.144	1.013 to 1.292	0.030
Black vs. API	3.040	2.562 to 3.607	<0.001	2.400	2.103 to 2.738	<0.001
**Age- and luminal subtype-adjusted**						
White vs. Black	0.333	0.304 to 0.364	<0.001	0.358	0.332 to 0.385	<0.001
White vs. API	0.999	0.851 to 1.721	0.987	0.952	0.843 to 1.077	0.436
Black vs. API	2.265	1.896 to 2.705	<0.001	2.051	1.791 to 2.349	<0.001
**Fully adjusted**						
White vs. Black	0.132	0.119 to 0.147	<0.001	0.254	0.235 to 0.274	<0.001
White vs. API	0.402	0.339 to 0.475	<0.001	0.689	0.609 to 0.780	<0.001
Black vs. API	0.916	0.749 to 1.121	0.396	1.276	1.106 to 1.473	<0.001

*BCSS, breast cancer-specific survival; OS, overall survival; APIs, asian or pacific islanders; HR, hazard ratio; CIs, confidence intervals*.

### Survival Analysis in Mastectomy Group

**Figure 3:**
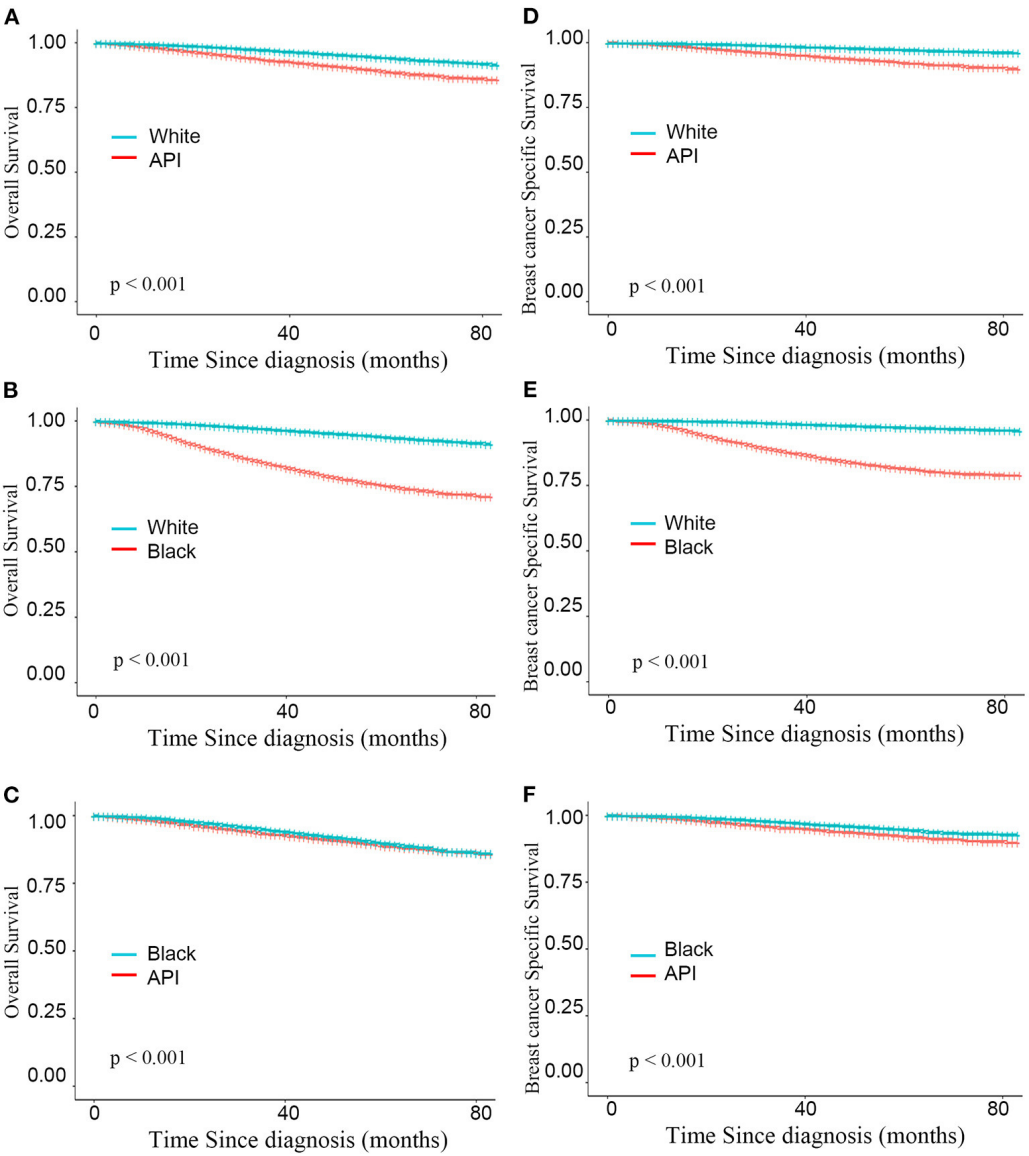
Kaplan–Meier curves for overall survival by race **(A–C)** and breast cancer-specific survival **(D–F)** based on the inverse probability weighting of all factors in mastectomy group.

As shown in Figure 3, after adjusting the imbalanced clinicopathological characteristics in different races, the white individuals after mastectomy also had the longest survival was higher than that of the minority groups (BCSS: HR_Whitevs.API_ = 0.325, HR_Whitevs.Black_ = 0.128; *P* < 0.001; OS: HR_Whitevs.API_ = 0.481, HR_Whitevs.Black_ = 0.206; all *P* < 0.001). Interestingly, compared with BCS group, the survival advantages of whites were declined in mastectomy group (BCSS:ΔHR_Whitevs.API_ = 19.15%, ΔHR_Whitevs.Black_ = 3.03%; OS:ΔHR_Whitevs.API_ = 30.19%, ΔHR_Whitevs.Black_ = 18.90%). Detailed SHR results are given in Table 4.

**Table 4:** Inverse probability weighted (IPW) estimates of BCSS and OS in patients underwent mastectomy.

**Ref**	**BCSS**			**OS**		
	**HR**	**CIs**	***P***	**HR**	**CIs**	***P***
**Un-adjusted**						
Whites vs. Blacks	0.568	0.532 to 0.607	<0.001	0.669	0.633 to 0.707	<0.001
Whites vs. APIs	1.498	1.350 to 1.663	<0.001	1.696	1.554 to 1.851	<0.001
Blacks vs. APIs	2.636	2.346 to 2.960	<0.001	2.534	2.296 to 2.796	<0.001
**Age-adjusted**						
Whites vs. Blacks	0.544	0.509 to 0.582	<0.001	0.497	0.469 to 0.527	<0.001
Whites vs. APIs	1.409	1.269 to 1.565	<0.001	1.183	1.081 to 1.295	<0.001
Blacks vs. APIs	2.630	2.341 to 2.953	<0.001	2.279	2.061 to 2.519	<0.001
**Age and luminal subtype adjusted**						
Whites vs. Blacks	0.423	0.395 to 0.453	<0.001	0.405	0.381 to 0.430	<0.001
Whites vs. APIs	1.091	0.982 to 1.213	0.106	0.954	0.870 to 1.046	0.313
Blacks vs. APIs	2.056	1.823 to 2.319	<0.001	1.843	1.661 to 2.405	<0.001
**Full adjusted**						
Whites vs. Blacks	0.128	0.118 to 0.139	<0.001	0.206	0.193 to 0.707	<0.001
Whites vs. APIs	0.325	0.290 to 0.365	<0.001	0.481	0.436 to 0.532	<0.001
Blacks vs. APIs	0.635	0.546 to 0.738	<0.001	0.862	0.765 to 0.971	<0.001

*BCSS, breast cancer specific survival; OS, overall survival; APIs, asian or pacific islanders; HR, hazard ratio; CIs, confidence intervals*.

## Discussion

Based on a large multiracial dataset, this study found that there were distinct racial disparities in the survival of breast cancer patients after surgery. In the clinicopathological characteristics unadjusted dataset, API patients had the best BCSS and higher OS than patients of other races. However, after adjusting for the imbalanced clinicopathological features in different races by IPW, white patients had a better prognosis than minority patients regardless of surgery types. Noteworthy, we found the survival differences between whites and minorities were increased in mastectomy group.

In the past century, the total mastectomy proposed by Halsted was regarded as the standard surgery for breast cancer patients with localized cancer (13). However, this surgery was too radical to improve the quality of life of patients (14). It was gradually phased out in several large randomized clinical trials, and medical development brought less harm and higher quality of life (8, 15, 16). At 5, 8, 12, and 20 years of follow-up, initial reports of the NSABP B-06 trial consistently indicated that breast cancer patients who underwent segmental mastectomy with or without breast irradiation had no worse BCSS and OS outcomes than patients with total mastectomy (15, 17–19). Unexpectedly, BCS patients after radiotherapy even had better survival than those with total mastectomy at the 20-year follow-up (15). In fact, clinical trials and long-term follow-up analyses demonstrated that there was no worse survival between BCS and total mastectomy patients (20–23), indicating that BCS was a standard surgery for breast cancer patients in the early stage rather than total mastectomy. However, compared with mastectomy, BCS relies on precise imaging, detailed pathology, specialist surgery, and systemic therapy to prolong survival (9). The sociodemographic disparities in different races may lead to survival disparities in patients receiving different surgery types.

Although previously studies deliver evidence encouraging the use of BCS, it remains unclear why such survival disparities would exist in breast cancer patients with the same stage. In fact, BCS is a complex treatment, which relies on precise imaging and pathology, specialist surgery, and systemic therapy to enhance survival (9). Factors of ethnic heterogeneity, resulting in a worse prognosis in minorities, include younger age at diagnosis, later stage of breast cancer at diagnosis, biologic and genetic factors (24). However, in the current study, we found that adjusted for imbalanced clinicopathological features, such as age at diagnosis, grade, stage, and luminal subtype, minorities breast cancer patients still had a worse prognosis than white breast cancer patients. Thus, in addition to the adjusted clinicopathological features, several racial traits, including biologic and genetic factors, health care, and socioeconomic status, tended to affect the survival of breast cancer patients. For example, compared with white women, black women were less likely to receive appropriate treatment. Budhwani et al. (24) found that black women were less likely to receive surgical treatment in tertiary hospitals and did not receive adjuvant therapy in a timely manner. Interestingly, we found that API patients had a better prognosis than those from other races in the unadjusted data because they were less likely to be diagnosed at the T4 and N3 stages before BCS, suggesting that BCS should only be performed in APIs at early stages for a better prognosis.

In this study, we also found that in the mastectomy group, white breast cancer patients still had a better prognosis than minorities breast cancer patients. These results suggest that factors beyond clinicopathological features also affect the survival of breast cancer patients after mastectomy. Interestingly, we found that comparing the survival differences between white and minority breast cancer patients after BCS, the survival differences were increased in patients after mastectomy. These phenomena may be attributed to several unmeasurable factors, including socioeconomic status, multimorbidity, and effective therapy after surgery. For example, mastectomy is more common in women with a lower socioeconomic status, higher multimorbidity, and lower rates of adjuvant chemotherapy (25–27).

There were inherent limitations in these cancer registry datasets. First, owing to data availability, other information from SEER could not be collected, such as surgical distance, income, premenopausal endogenous hormone levels, breastfeeding, and genetic susceptibility, so it is hard to adjust for all potential confounders in this study (28–33). Second, white patients had higher survival rates, which may be influenced by adjuvant chemotherapy and radiotherapy after surgery. However, cancer registries did not collect treatment regimens systematically after surgery. In this study, the rates of radiotherapy were similar among individuals of these three races. Moreover, black patients with the worst survival rates even had the highest rate of adjuvant chemotherapy compared with women from other races. Despite limitations, this study offered a perspective on the racial disparities of breast cancer patients based on a large-scale multiracial dataset. Additionally, it adjusted for the imbalanced baseline measured by the IPW method before the analysis of cumulative racial disparities to reduce the probability of confounding.

In conclusion, we first identified that minority breast cancer patients had worse survival outcomes than white patients regardless of patients clinicopathological features and surgery types. The results suggested that survival disparities in breast cancer patients may not only be explained by the disparities of clinicopathological characteristics, but also affected by other ethnic differences. Confirming these risk factors through further research could improve the prognosis of breast cancer patients.

## Data Availability Statement

The original contributions presented in the study are included in the article/supplementary materials, further inquiries can be directed to the corresponding authors.

## Ethics Statement

Ethical review and approval was not required for the study on human participants in accordance with the local legislation and institutional requirements. The patients/participants provided their written informed consent to participate in this study.

## Author Contributions

Conceptual construction and project administration by JL and SH. Data collection and compilation by SW and WT. Data analysis and interpretation by JL. Writing by SW, WT, and JL. Visualization by SH. Supervision by SW. All authors contributed to the article and approved the submitted version.

## Funding

This study was supported by National Natural Science Foundation of China (81802641).

## Author Disclaimer

The authors are responsible for this study, which does not represent the views of the National Institutes of Health in the United States.

## Conflict of Interest

The authors declare that the research was conducted in the absence of any commercial or financial relationships that could be construed as a potential conflict of interest.

## Publisher's Note

All claims expressed in this article are solely those of the authors and do not necessarily represent those of their affiliated organizations, or those of the publisher, the editors and the reviewers. Any product that may be evaluated in this article, or claim that may be made by its manufacturer, is not guaranteed or endorsed by the publisher.
